# Artemether–lumefantrin treatment adherence among uncomplicated *plasmodium falciparum* malaria patients, visiting public health facilities in AsgedeTsimbla district, Tigray, Ethiopia: a cross-sectional study

**DOI:** 10.1186/s13756-020-00846-y

**Published:** 2020-11-10

**Authors:** Mekonnen Gebremichael Gebrekidan, Gebretsadik Berhe Gebremedhin, Yosef Sibhatu Gebregiorgis, Alefech Addisu Gezehegn, Kissanet Tesfay Weldearegay

**Affiliations:** 1 Tigray Regional Health Bureau, Mekelle, Tigray Ethiopia; 2 College of Health Sciences, School of Public Health, Department of Epidemiology, Mekelle University, Mekelle, Tigray Ethiopia; 3 Federal Ministry of Health, Mekelle, Tigray Ethiopia

**Keywords:** Artemether–lumefantrine, Adherence, *Plasmodium falciparum*

## Abstract

**Background:**

Ethiopia has set a goal to eliminate malaria by 2030; Artemether–lumefantrine (AL) is put as one of the cornerstone strategies for uncomplicated *plasmodium falciparum* malaria treatment. However, only focusing on prescribing of the treatment without assessing patients’ adherence could lead to the resistance of the drug. In Ethiopia, there is limited evidence about patients’ adherence to AL and its influencing factors. Therefore, this study aimed at addressing this information gap.

**Methods:**

A health facility based cross-sectional study was employed. Participants were selected using simple random sampling technique from registration books of the public health facilities in AsgedeTsimbla. Data were collected from March 24th to April 30th, 2018. We interviewed participants using a pre-tested structured questionnaire, and the blister pack was also inspected at their homes on day 4. Data were entered into Epi-Info and analyzed using SPSS 21. Odds ratios with 95% Confidence Intervals were estimated and the level of significance was declared at p-value ≤ 0.05.

**Results:**

A total of 384 study participants were interviewed with a response rate of 95.5%. The overall AL adherence was 53.6% (95% CI 48.4–58.3%). Children aged < 5 years [AOR: 0.4, 95% CI (0.2–0.8)], and being treated in health post [AOR: 0.3, 95% CI (0.1–0.5)] were more likely to show AL adherence whereas illiteracy [AOR: 9.4, 95% CI (4.2–21.3)], didn’t know the consequence of discontinued AL [AOR: 4.0, 95% CI (2.1–7.6)], had concomitant drugs [AOR: 2.5, 95% CI (1.4–4.5)], and stopped/saved drug when improved before tablet got finished [AOR: 3.2, 95% CI (1.7–5.9)] were factors less likely to be associated with AL adherence.

**Conclusion:**

AL adherence was low. Children aged < 5 years, and being treated in health post were determinants of AL adherence whereas illiteracy, didn’t know the consequence of discontinued the drug, had concomitant drugs, and stopped/saved drug when improved before tablet got finished were factors that hindered the AL adherence. Stakeholders should emphasize designing appropriate strategies including educational interventions to increase the AL adherence and prevent drug resistance. Further research should be conducted to evaluate AL resistance.

## Introduction

In 2001, the World Health Organization (WHO) recommended artemisinin-based combination therapy (ACT) including Artemether–lumefantrine (AL) as a first-line treatment for uncomplicated *Plasmodium falciparum (PF)* for all countries that experienced resistance to mono-therapies, notably to chloroquine and sulfadoxine-pyrimethamine (SP) [1]. After three years (2004), Ethiopia adopted AL as a first line treatment following the widespread resistance of SP in the country. In 2017, AL plus single dose of primaquine was recommended as first-line drugs [2–4]. AL is co-formulated tablets of 20 mg of Artemether and 120 mg of Lumefantrine (Coartem®; Novartis) taken twice daily for the subsequent three days; respecting strict eight hour interval between the first and the second dose; also, each dose should be accompanied by fatty meal to maximize absorption. The drug is dosed according to weight or age and supplied in blister packs containing 1–4 tablets. While the artemisinin quickly reduces most of the parasites load, the partner drug clears the remaining ones. If the patient fully adhered, AL has 98% efficacy [3, 5, 6].

Adherence refers to the extent to which a person takes the prescribed drugs as directed; therefore, adherence to AL is a crucial part of patient care and indispensable for reaching clinical goals. Increasing patient’s adherence to AL may have so far greater impact on the health of the population than any improvement in specific medical treatment. In contrast, not adhering to this drug leads to poor clinical outcomes, increased morbidity and mortality, and unnecessary healthcare expenditure. Eventually, it might lead to the emergence and spread of drug resistant *PF* malaria strains [3, 7, 8]. Patients with uncomplicated *PF* malaria have strongly discontinued the full or partial course of AL when they were symptom-free since its symptoms improve rapidly after the treatment was initiated [9–12]. Multiple factors might lead to poor AL adherence, normally classified into five categories: socioeconomic factors [12–16], patient-related factors [11–13, 17–20] drug and condition-related factors [14, 21], and health system-related factors [13].

Malaria is preventable and treatable, but if the patient does not adhere to treatment, it leads to severe malaria that has a case fatality rate of 10–20% [22, 23]. The serious consequences of non-adherence to AL implies not only the social and health costs of treatment failure at the patient level but also at national and global level, where resistance to AL has had a significant impact on the cost of malaria control due to the need for a new drug [24, 25].

Globally, non-adherence to anti-malaria treatment has been identified as a key factor for poor clinical outcomes and is one of the greatest challenges to malaria control efforts today [26].

Importantly, the mechanism behind the development and spread of SP-resistant *PF* strain was a complex one with multiple factors, but non-adherence to SP treatment was the main reason [27, 28].

Therefore, to keep AL efficacy, patient’s adherence to the drug is crucial. Thus, assessing the magnitude and determinants of optimal AL adherence is urgently needed to develop effective intervention strategies for achieving the national malaria elimination goal in the stated period [25].

Concerning this specific public health problem in Ethiopia, as per our knowledge, there is little knowledge and available evidence on the raised public health problem in Ethiopia. This study aimed at assessing patients’ adherence with AL and identifying its influencing factors in Asgede Tsimbla district, Tigray, Ethiopia.

## Methods and materials

### Study setting, design, and population

This study was conducted in Asgede Tsimbla district which is a predominantly rural area located in the northwestern of Tigray, Ethiopia. It is administratively sub-divided into 25 rural and two urban *kebelles* (smallest local administrative unit). The entire district is a malarious area which is characterized by the climatic factors that determine the malaria endemic area including, the average temperature range of 25 to 35 °C, altitude of < 2000 m above sea level, and annual rain fall of 500–900 mm [29]. The healthcare system consists of 7 health centers (provide inpatient and outpatient curative care services, and malaria is diagnosed using microscope by laboratory technician/technologist) and 15 health posts (provide outpatient curative care services with more focus on preventive activities, and malaria is diagnosed using Rapid Diagnostic Test, RDT by health extension workers). All public health facilities in the district distributed AL free of charge. Six health centers and eight health posts that had reported *PF* malaria in the previous month (March) were selected and included in the study. The study period was from 24th March to 30th April, 2018.

A health facility based cross-sectional study was employed to assess the magnitude of AL treatment adherence and its determinants among uncomplicated *Plasmodium falciparum* malaria patients.

People who experienced uncomplicated *PF* malaria, and treated with AL in all public health facilities of AsgedeTsimbla district were considered as source population; however, those patients who visited the respected health facilities for seeking treatment during the study period were considered as study population and finally, patients who diagnosed with uncomplicated malaria and started AL treatment were randomly selected from the studied health facilities.

## Eligibility criteria

### Inclusion criteria

A registered confirmed uncomplicated *PF* malaria patients at the selected health facilities who were permanent residents were eligible for the study.

### Exclusion criteria

A patient who could not talk or listen, and/or who had a mental problem or was critically ill.

### Sample size determination, sampling techniques and procedures

#### For specific objective one

The sample size was computed for a single population proportion formula using; $\mathrm{n}= {\left(\mathrm{Z}|\frac{\mathrm{\alpha }}{2}\right)}^{2}*P(1-P)/{W}^{2}$ with the following assumptions; 38.7% of adherence to AL from previous study [12]; 95% confidence interval, 5% margin of error, and adding 5% non-response rate and 5% lost to follow up, the final computed sample size was 402.

#### For specific objective two

Factors associated with adherence to AL; the sample size was computed using Epi Info software version 7.2.1 with consideration of consistent determinants of adherence to AL reported in the literature: taking 1st dose at health facilities, vomiting, and packaging information for prescribed drug instructions with the following assumptions: 95% CI, 80% power, ratio of 1:1. Therefore, the calculated sample sizes for the selected pertinent factors were 250, 168 and 120, respectively. Finally, the study used the exclusive sample size of 402.

**Fig. 1:**
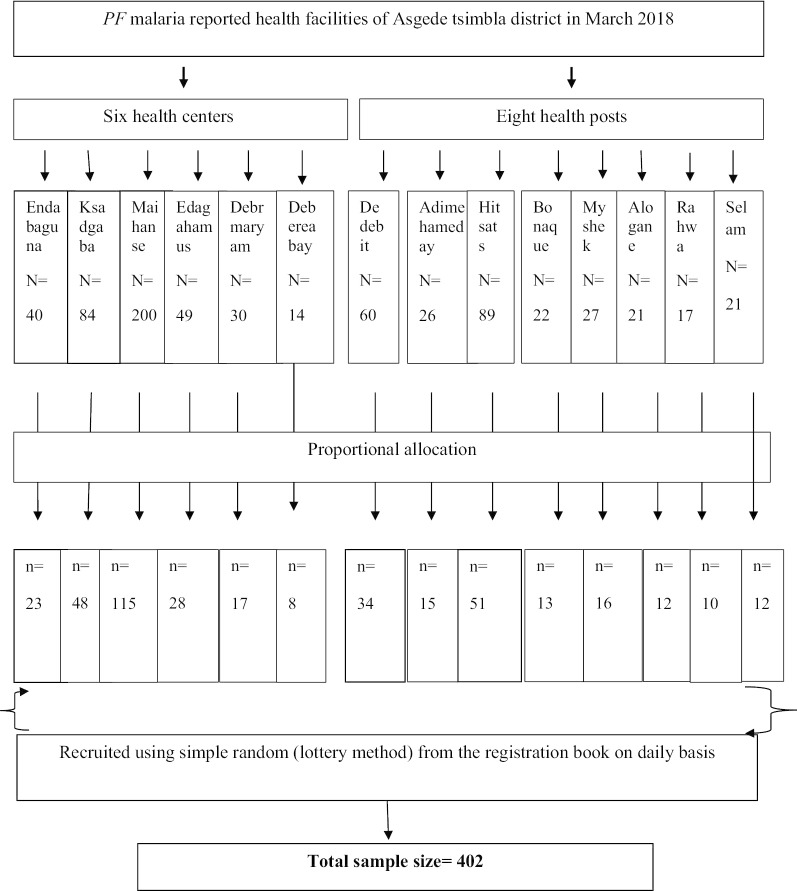
Schematic presentation of sampling procedure among uncomplicated *PF* malaria patients in AsgedeTsimbla district, Tigray, Ethiopia, 2018

Firstly, all public health facilities of the district which had reported *PF* malaria cases in the preceding month (March) were included in the study. Secondly, 6 out of 7 health centers and 8 out of 15 health posts were selected to have representative sample using simple random sampling technique. The next step, the required sample size was allocated using proportional to size technique based on a month of March 2018 malaria case report of the studied health facilities. Finally, the actual study participants were selected using simple random sampling; however, all the registered under-five children who were diagnosed with the disease and received AL treatment were totally included during the study period. In the situation where more than one patient sought treatment from respective health facility in a day from the same household; one of them was selected randomly (Fig. 1).

## Data collection tools and methods

The questionnaire tool was adapted from relevant literature [12, 13, 30]. The study used a structured and pre-tested questionnaire for data collection after translation to the local language (Tigrigna). It also used checklist to inspect the remaining tablet in the blister pack.

An interviewer-administered questionnaire was used to collect the data from March 24 to April 30/2018. On day zero, tracing address was collected, and then patients were visited at their house on the day after AL treatment was supposed to be complete (Day 4). Data were collected by six trained clinical nurse diploma professionals and three BSc. nurses were also assigned as supervisors. Adherence was determined using two different methods: pill count and interview. The availability of blister pack was used to present the visual pill count inspection and to checked out the remaining AL tablet on the pack. The data collectors collected relevant data as per the questionnaire tool under strict supervision. The adherence to AL was determined using pill count only; dose and dose timing interview recall only, and both pill count and interview approaches.

The number of patients recruited each day by data collector was limited to a maximum of five because tracing of the study participants at their household was difficult and time-consuming. Adult patients or caregivers for children patients who were not available in their home on the day of the visit were revisited on the next day and if not found at the second visit, they were considered as non-response.

Timing of medication was assessed by considering natural events with local expression, such as the position of the sun, coffee time/cow milking, time from church and time of cattle leaving or entering their shed. We converted these events to approximate hours. Therefore, the time interval for the dose was considered correct if taken ± 2 h. from the expected time when it was supposed to be taken [12, 13].

### Data quality control

The questionnaire was pretested in 5% of participants who were not part of our study to ensure its validity and appropriateness in the local context. Moreover, pre-testing findings such as skip pattern and sequencing of questions were incorporated according to the pre-testing findings. Data collectors and supervisors were trained for two days on how to approach respondents, ethical issues, how to fill the questionnaire using mock exercises and observation forms**.** Three pharmacy technicians reviewed the questionnaire prior to actual data collection.

At the end of each day, the principal investigator and the supervisors checked out the consistency and completeness of the filled questionnaire. If not, the data collectors went back to the field to complete the questionnaire. The health workers who were responsible for the diagnosis and treatment of malaria did not participate in the study, and the patients/caregivers didn’t know that they would be visited at their household.

## Study variables

### Dependent variable

Artemether–lumefantrine adherence status (having taken all the medication as prescribed; at correct time, amount, no spitting or vomiting within the first half an hour and if happened, dose re-administered and empty blister pack; otherwise non-adherent to AL).

### Independent variables

#### Socio-demographic factors

Age, sex, education, residence, and income status.

#### Patient-related factors

Having a radio, treatment-seeking behavior, preference route of AL, knowing dose and side effect of AL, vomiting as an experienced side effect, knowing the consequence of not finished treatment, beliefs on traditional treatment, belief on the severity of malaria, attitudes towards the sharing and saving of AL.

#### Health worker related factors

Giving clear instruction, 1st dose given at health facility (HF), provided a chance to repeat the instruction, and technique of instruction given.

#### Drug or condition related factors

worsening or improved condition, vomiting after taking the drug, unpleasant/ bad tastes, too many tablets to take, and concomitant treatment.

## Data processing and analysis

The completeness and accuracy of the collected data were checked then data were coded, entered and cleaned with Epi Info version 7.2.1, and statistical analyses were performed using SPSS version 21.0. Descriptive analyses were conducted and generated frequencies with percentages and charts for categorical variables, and median with inter-quartile range after checking normality for continuous variables. A binary logistic regression model was fitted and logistic regression analyses at bivariate and multivariable levels were estimated to determine the effect of the different independent variables on the outcome variable using Crude Odds Ratio (COR) and adjusted Odds Ratios (AOR) with their corresponding 95% Confidence Intervals (CIs).

To obtain potential independent variables and avoid unstable estimate in the final model, only variables from the bivariate analysis that had a p-value of ≤ 0.25 were considered as candidate independent variables in the multivariate analysis to control the effects of confounders. P-values of ≤ 0.05 in a multivariate logistic regression were considered as statistically significant association with adherence to AL. Multi-collinearity using variance inflation factor (VIF), chi-square test assumptions, and model fitness using Hosmer and Lemeshow goodness-of-fit-test statistics were checked.

### Operational definitions

#### Un-complicated *PF* malaria

Patients having *PF* or mixed infection (*Plasmodium falciparum* and *Vivax*) with RDT or microscopy, without signs of severity or evidence of vital organ dysfunction, and with or without the following manifestations, fever, chills, rigors, headache, body pains, nausea, vomiting, and joint weakness, and in physical examination may reveal pallor and hepatosplenomegaly [4].

#### Definition of adherence

Adherence was classified according to the findings from the blister pack, and/or the response to the interview of the participants; if there is discrepancy between blister packs and self-report, we primarily used the former as confirmatory one.

#### Adherent

If patient reported, having taken all the medication as prescribed, on the correct time and interval and with the correct amount, and with no spitting or vomiting within the first half-hour, or when such spiting/vomiting dose has re-administered, and/or the blister packaging presented was empty; however, if he/she could not take the drug based on the above prescription instructions, this marked him/her as non-adherent to AL [12, 13, 31].

#### Incorrect intake

Having not taken all the medication at the correct time and interval.

#### Incomplete intake

Having not taken all the medication as prescribed, with the correct amount, and/or with spitting or vomiting within the first half-hour, or when such spiting/vomiting dose has not been re-administered.

#### Knowing consequence of not finishing AL

If a patient reported; either as negative treatment outcome, and/or drug resistance as a consequence of not finished AL as recommended.

#### Recall side effects

If the patient reported at least one of the following; headache, vomiting, and arthralgia and weakness as a side effect of AL [3].

## Results

### Socio-demographic characteristics of the study participants

Of the 402 participants selected, 384 (95.5%) were successfully visited, while 4.5% were lost to follow up, and none of the participants refused to participate. The median with interquartile range (IQR) of study participants’ age was 17 ± 29.7 years which ranged from 1 to 74 years. One hundred sixty one (42%) study participants were illiterate (Table 1).

**Table 1:** Demographic and socio-economic characteristics of uncomplicated *P. falciparum* malaria patients in AsgedeTsimbla, Tigray, Ethiopia, 2018 (n = 384)

Patients' characteristics		Frequency n (%)
Sex	Male	174 (45.3)
	Female	210 (54.7)
Age group (years)	< 5	96 (25)
	5 to 17	96 (25)
	≥ 18	192 (50)
Marital status	Married	168 (43.8)
	Single	32 (8.3)
	Divorced/Widowed	32 (8.3)
	Underage (< 18 yeras of age)	152 (39.6)
Religion	Orthodox Christian	368 (96)
	Others*	16 (4)
Educational level	Illiterate	161(41.9)
	Primary (1–8 grade)	123 (32.1)
	Secondary and above	100 (26)
Residency status	Semi-urban	214 (55.7)
	Rural	170 (44.3)
Occupational status	Farmer	184 (48)
	Merchant	70 (18.2)
	Government employee	66 (17.2)
	Private employee	64 (16.6)
Had radio/TV	Yes	226 (58.9)
	No	158 (41.1)
Race	Tigrayan	376 (98)
	Others**	8 (2)
Household size	< 5	232 (60.4)
	≥ 5	152 (39.6)

*Others: Catholic, Protestant; **Others: Amhara, Oromo

### Adherence to Artemether–lumefantrine treatment (AL)

Out of the 384 patients interviewed, 206 [53.6% (95% CI 48.4–58.3%)] adhered to AL treatment. All these 206 patients who were adherent to AL were evaluated using both pill count and dosage timing approaches; therefore, 37.4% (77/206), 29.6% (61/206) and 33% (68/206) patients were labeled as adherent for pill count only, for dosage timing, and both pill count and dosage timing, respectively.

**Fig. 2:**
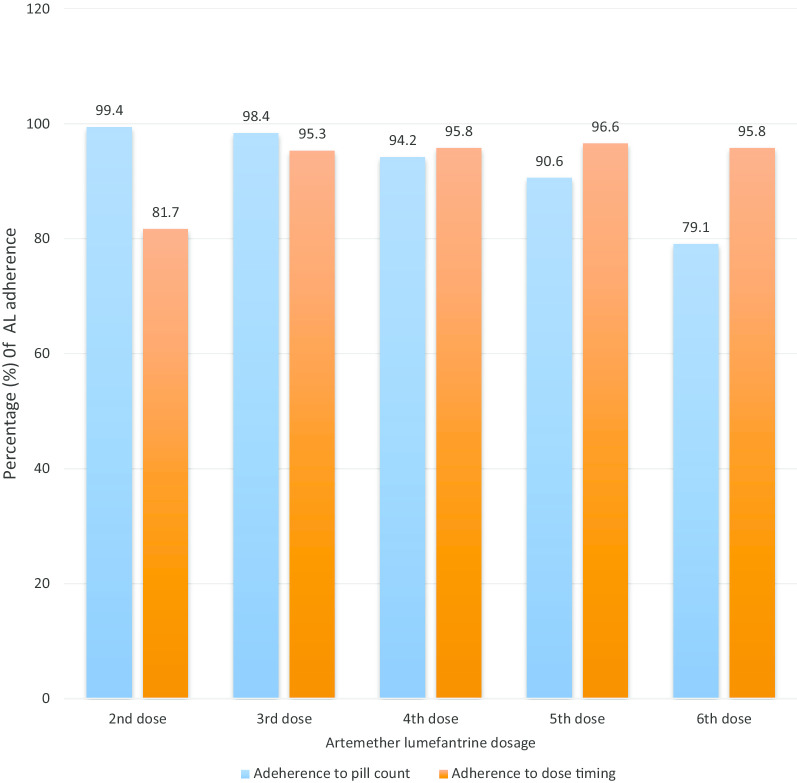
Pill count and dose timing adherence to AL treatment among uncomplicated PF malaria patients in AsgedeTsimbla district, Tigray, Ethiopia, 2018

Regarding observational findings during the household visit; the blister pack was shown by 75.8% (291/384) of patients, of these, 28.8% (84/291) had one or more pills left on the blister pack (day 3). Of the 36 patients who vomited the drug within half hour of ingestion, 52.6% had re-administered the dose, but did not seek for the replenishment of the dose, while 47.4% opted to wait for the next dose. Out of the 138 (77forpillcountonlyand61fordosagetiming) who experienced adherence to AL treatment, almost the same proportion of malaria patients (80%) were adherent according to pill count and dose timing at the 2nd and 6th doses whereas the remaining 3^rd^, 4th and 5th AL doses were appropriately taken by majority of malaria patients (90%) who experienced pill count and dose timing adherence to AL treatment (Fig. 2).

The two main reported reasons for the incomplete intake of AL were “felt better before the treatment course finished” (27%) and “simply forgot” (19.3%) whereas the most common reasons for incorrect intake were “the instruction was not understood” (50.4%) and “simply forgot” (27.8%) (Table 2).

**Table 2:** Reasons of incomplete and incorrect intake of Artemether–lumefantrine treatment among uncomplicated *P. falciparum* malaria patients in AsgedeTsimbla district, Tigray, Ethiopia, 2018 (n = 178)

	Frequency n (%)
*Why tablet left over (incomplete intake)*	
Felt better before treatment course finished	34 (27)
Simply forgot	24 (19)
Shared with others	16 (12.7)
Vomited/repeated after vomiting	16 (12.7)
Side effects experienced	11 (8.7)
Tablets had bitter taste	10 (8)
Too many tablets	8 (6.3)
Not improved	4 (3.2)
Alcohol drunk	3 (2.4)
*Why tablet was taken incorrectly (dose timing)*	
Did not understand the instruction	58 (50.4)
Simply forgot	32 (27.8)
Side effects experienced	12 (10.4)
Being out-of-home for long time	13 (11.3)

### Patients’, drug and condition, and health facility/workers’ related characteristics

Two-thirds of the study participants, 295 (76.9%) reported that when they suspected malaria symptoms, they sought treatment from nearby public health facilities. Less than half, 188 (49%) of study participants perceived tablet as a preferred drug formulation. This study also revealed that a majority (90%) of patients showed improvement during the household visit.

Regarding drug and condition related characteristics, 264 (68.8%) of study participants did not know that AL was taken with fatty food or milk. Two hundred eighty-five (74%) patients reported that they did not get a chance to repeat the instruction about the AL prescribed information. Only 58 (15.1%) of patients took their first AL dose at the dispensing room of the health facility. Less than half, 176 (45.8%) patients were provided package information for the prescribed drug (Table 3).

**Table 3:** Patients’, drug and condition, and health facilities/workers’ related characteristics among uncomplicated *P. falciparum* malaria patients in AsgedeTsimbla, Tigray, Ethiopia, 2018 (n = 384)

Variable	Frequency n(%)
*Patients’ related characteristics*	
*Suspected malaria symptoms*	
Sought treatment at health facility	303 (78.91)
Purchased drug from a drug shop	77 (21.08)
Used herbal medicine	4 (0.01)
*Knew malaria was a severe/fatal disease*	
Yes	284 (73.9)
No	100 (26.1)
*Have you ever used AL treatment*	
Yes	288 (75)
No	96 (25)
*Knew Coartem® treated malaria effectively*	
Yes	342 (89)
No	42 (11)
*Recalled correct AL dose*	
Yes	376 (97.9)
No	8 (2.1)
*Recalled correct AL dose timing*	
Yes	328 (85.4)
No	56 (14.6)
*Recalled AL side effects*	
Yes	80 (20.8)
No	304 (79.2)
*Perceived preference of drug formulation*	
Tablet	188 (49)
Syrup	112 (29.1)
Injection	84 (21.9)
*Drug and condition related characteristics*	
*Improved during the household visit*	
Yes	346 (90)
No	38 (10)
*Knew the consequences of not finishing AL treatment*	
Yes	132 (34.4)
No	252 (65.6)
*Had concomitant drugs*	
Yes	164 (42.7)
No	220 (57.3)
*Did AL treatment have too many tablets*	
Yes	214 (55.7)
No	170 (44.3)
*Had AL bitter taste*	
Yes	202 (52.6)
No	182 (47.4)
*Vomited within 30 min of ingestion*	
Repeated the dose	196 (51)
Waited until the next dose	188 (49)
*Improved before tablets got finished*	
Continued until the full dose was finished	238 (62)
Stopped the medication/saved tablet for future	146 (38)
*Worsened before tablets got finished*	
Returned to a health facility	304 (79.2)
Continued until tablets got finished	39 (10.1)
Used herbal medicine	26 (6.7)
Took more dose than prescribed	15 (3.9)
*Knew AL was taken with fatty food or milk*	
Yes	120 (31.2)
No	264 (68.8)
*Health facilities/workers related characteristics*	
Treatment received	
At health center	222 (57.8)
At health post	162 (42.2)
*The first dose of AL was taken at*	
Home	326 (84.9)
Health facility	58 (15.1)
*Got instruction on how to take the drug*	
Yes	384 (100)
*Method of instruction given*	
Verbal only	158 (41.1)
Verbal and written	184 (47.9)
Package was used as a visual aid	42 (10.9)
*Got a chance to repeat AL prescription instruction*	
Yes	99 (25.8)
No	285 (74.2)

Coartem®: trademark symbol, *AL* Artemether–lumefantrine

### Determinant factors of adherence to AL

When the above fifteen candidate variables were fitted into multivariate binary logistic regression, only six factors, including children aged < 5 years, illiteracy, treatment received at health post, didn’t know the consequences of AL discontinued, stopped/saved drug when improved before tablet got finished, and had concomitant drugs were associated with AL non-adherence (Table 4).

**Table 4:** Bivariate and multivariate analyses factors associated with adherence to AL among uncomplicated *P. falciparum* malaria patients in AsgedeTsimbla district, Tigray, Ethiopia, 2018 (n = 384)

Variable		Adherence to AL		COR (95%CI)	P-value	AOR (95%CI)	P-value
		Non-adhered n (%)	Adhered n (%)				
Age group (years)	< 5	27 (15.2)	69 (33.5)	0.3 (0.1–0.52)	0.01	0.4 (0.2–0.88)	0.02*
	5 to 17	44 (24.7)	52 (25.2)	0.6 (0.4–1.1)	0.11	0.7 (0.3–1.41)	0.74
	≥ 18	107 (60.1)	85 (41.3)	1		1	
Educational level	Illiterate	108 (60.7)	53 (25.7)	15 (7.5–29.7)	0.01	9.4 (4.2–21.3)	0.01**
	Primary (1–8 grade)	58 (32.6)	65 (31.6)	6.5 (3.2–13.1)	0.01	4.5 (1.9–10.2)	0.01**
	Secondary and above	12 (6.7)	88 (42.7)	1		1	
Had radio/TV	No	86 (48.3)	72 (35)	1.7 (1.1–2.6)	0.01	0.8 (0.4–1.5)	0.5
	Yes	92 (51.7)	134 (65)	1		1	
Treatment given	Health post	56 (31.5)	106 (51.5)	0.4 (0.2–0.65)	0.01	0.3 (0.1–0.55)	0.01**
	Health center	122 (68.5)	100 (48.5)	1		1	
Improved during visit	No	6 (3.4)	12 (6)	0.5 (0.2–1.5)	0.26	0.9 (0.2–3.4)	0.87
	Yes	172 (96.6)	194 (94)	1		1	
Recalled side effect	No	160 (90)	144 (70)	3.8 (2.1–6.7)	0.01	1.1 (0.4–2.5)	0.8
	Yes	18 (10)	62 (30)	1		1	
Knew consequences of AL discontinued	No	174 (84.5)	78 (43.8)	6.9 (4.3–11.2)	0.01	4.0 (2.1–7.6)	0.01**
	Yes	32 (15.5)	100 (56.2)	1		1	
Had AL bitter taste	No	64 (36)	118 (57.3)	0.7 (0.4–1.0)	0.08	0.8 (0.4–1.4)	0.5
	Yes	114 (64)	88 (42.3)	1		1	
Vomited within 30 min of ingestion	Waited till the next dose	112 (62.9)	76 (36.9)	2.9 (1.9–4.3)	0.01	1.0 (0.5–1.9)	0.92
	Repeated the dose	66 (37.1)	130(63.1)	1		1	
Improved before tablets got finished	Stopped/saved drug	111 (62.4)	35 (17)	8.1 (5.0–13)	0.01	3.2 (1.7–5.9)	0.01**
	Continued till finished	67 (37.6)	171 (83)	1		1	
Perceived preferred drug formulation	Syrup	57 (32)	34 (16.5)	3.6 (2.1–6.1)	0.01	1.8 (0.9–4.2)	0.06
	Injection	67 (37.6)	55 (26.7)	2.6 (1.6–4.2)	0.01	2 (0.9–4.2)	0.06
	Tablets	54 (30.4)	117 (56.8)	1		1	
Concomitant drugs	Yes	98 (55)	47 (22.8)	4.1 (2.6–6.4)	0.01	2.5 (1.4–4.5)	0.01**
	No	80 (45)	159 (77.2)	1		1	
First dose taken	At home	158 (88.8)	168 (81.6)	1.7 (0.9–3.2)	0.05	0.5 (0.2–1.2)	0.13
	At health facility	20 (11.2)	38 (18.4)	1		1	
Prescribed drug instruction given	Package as visual aid	82 (46.1)	102 (49.5)	0.7 (0.4–1.0)	0.11	1.0 (0.3–3.1)	0.87
	Verbal with written	12 (6.7)	30 (14.6)	0.3 (0.1–0.73)	0.01	2 (0.7–5.9)	0.16
	Verbal only	84 (47.2)	74 (35.9)	1		1	
Got chance to repeat prescription instruction	No	151 (84.8)	134 (65)	3 (1.8–4.9)	0.12	1.8 (0.9–3.6)	0.06
	Yes	27 (15.2)	72 (35)	1		1	

*p-value < 0.05; **p-value < 0.01

## Discussion

This study revealed that the magnitude of adherence to AL treatment was low, 53.6% (95% CI 48.4–58.3%). Age group < 5, and being treated in health post were factors associated with AL adherence whereas illiteracy, didn’t know the consequences of AL discontinued, stopped/saved drug when improved before tablets got finished, and had concomitant drug were factors that hindered the AL adherence after adjusting potential confounders. Adherence to AL is a key public health practice in attaining effective implementation of malaria case management strategy and prevention of AL resistance. The study revealed that significant proportion of uncomplicated malaria patients didn’t comply to AL treatment protocol including around one-third of patients didn’t finish their course of AL treatment on day 3 visit, half of patients who experienced vomiting didn’t re-administer and sought replenishment of missed dose, and just waited until the next dose. These findings show that the clinical and public health practices regarding case management and malaria elimination strategy have been implemented traditionally. Healthcare system of developing countries is challenged by a combination factors, poor socio-economic status, reduced availability and accessibility to health services, political issues as well as poor planning and/or poor implementation of health policies and programmes; nearly half of the study participants in the present study were illiterate, three-fourths didn’t get a chance to repeat the prescription instruction and a very small number of patients took their first dose under the direct observation of the dispenser. This indicates that healthcare actors (policy makers, planners, managers, and healthcare professionals) didn’t give more emphasis for provision of standard healthcare practices and minimizing early *PF* resistance.

This low rate of adherence level to AL treatment could be a big challenge to achieve the malaria elimination goal. The magnitude of adherence to AL treatment is consistent with the studies conducted in Ghana (57.3%), DRC (62%), and Kenya (60%) [18, 21, 32]. The present study had similar characteristics to cited comparable studies; both study settings were from rural and malaria endemicity with farming as the main source of income and low educational level of the participants. They also used the same outcome measurements (pill count and interview). However, the magnitude of the adherence to AL treatment in the current study was lower compared to studies done in Myanmar (85.7%), Tanzania (74.5% in public health facilities and 69.8% in retailers), and Malawi (65%) [13, 14, 33]. But it was higher than the study conducted in Tigray, Ethiopia (38.7%) [12], and in Ghana (36.6%) [17]. The disparities with the current study finding could be due to different methodological approaches in the previous studies, for instance, in the study of Maynamar, adherence level was classified into three categories: definitely non-adherent, probably non-adherent and probably adherent whereas in the current study, it was classified as adherent and non-adherent. In the study of Malawi, patients were informed that there would be a follow-up visit that could increase patients’ adherence. Moreover, in the study of Tanzania, patients residing within 2.5 km of the dispensary were included whereas in the present study, all patients were included without distance restriction, when the distance from dispensary increases patients’ adherence to AL decreases [25]. The sample sizes of studies from Ethiopia (n = 195), and Ghana (n = 175) were much smaller than the sample size of the current study (n = 384); this might have caused differences.

In the present study, two out of the five patients didn’t adhere to AL treatment. As the Ethiopian national malaria case management training manual indicates, *PF* parasites are only killed when the full course of the treatment is taken [4]; various methods have been used to measure the level of AL adherence, but none is fully satisfactory; however, adherence rate greater than 95% is mandatory particularly for acute diseases like malaria [34]. Non-adherence to AL treatment contributes to the recrudescence of malaria cases, affects clinical and parasitological cure rate, increases transmission rate, and eventually leads to the emerging of AL resistant *PF* parasite. This could also challenge achievement of the country’s malaria elimination goal on the stated time; therefore, there is a potential need for interventions to improve patient adherence with AL treatment.

Nearly eighty percent of non-adherent patients had not taken either 5th or 6th dose. Previous studies showed that doses 5 and 6 contribute most substantially to elevating Lumefantrine day-7 concentrations to levels sufficient to clear all *PF* parasites [6, 27]. Therefore, skipping the last 2 doses could increase the likelihood of recrudescence which in turn could lead to drug resistance.

This study revealed that age group < 5 years were about 60% less likely to experience non-adherence to AL as compared to age group ≥ 18 years. The possible explanation could be due to that children can be monitored by their parents’ to adhere to anti-malarial medication, thereby bring about higher adherence level. This finding is in line with the study done in Garrisa, Kenya in 2015 [32]. However, this result is inconsistent with the finding of a study from Malawi [13]. This disparity might be explained by the fact that the current study was carried out after the successful introduction of a specially created pediatric formulation of AL [35] whereas the former study was conducted before. This could be the reason for the acceptability and adherence to AL in young children in the current study [3].

Patients who were illiterate were 9 times more likely to experience non-adherence compared to those who attended secondary and above. This is in line with several studies done in sub-Saharan Africa; in Uganda [11], Kenya, and Tanzania [19, 33]. Formal education affects the patients’ understanding instructions, the quality of the patients’ relationship with the healthcare provider, and the ability of the patients to interpret the pictorial instructions and those written on the AL pack [15]. This study showed that patients who were being treated in health posts were about 70% less likely to be non-adherence as compared to those who were being treated in health centers. A study from Malawi indicated that non-adherence rate showed a significant difference across health facilities [13]. This could be due to that in health posts malaria diagnosis and treatment is given by health extension workers so that they may have more time to spend with each patient to explain details of the treatment schedules, and importance of adhering than the pharmacy technicians who are busy in the health centers. This may improve understanding of AL administration instructions and the importance of adhering in those patients who were being treated in health posts.

In this study, patients who didn’t know the consequence of incomplete treatment were about 4 times more likely to experience non-adherence compared to their counterpart. The present study finding was in agreement with the study done in Ghana [18]. The possible explanation could be that if patients do not know that they would be cured, and the emergence of drug resistance would be prevented if and only if the full dose is taken then they would adhere less. This appears to indicate that there was a poor understanding of the importance of finishing AL.

In our study, patients who reported to stop/save treatment if improved before tablets got finished were 3 times more likely to experience non-adherence compared to those who reported continuing till tablets got finished. Since *PF* malaria symptoms improved rapidly with AL initiation; there is a strong temptation not to complete the three-day course of AL [24, 36]. Patients who anticipate frequent malaria infections were more likely to be non-adherent, suggestive that the decision could be related to the desire to keep pills for the next malaria episode [25, 37]. This indicated that there was poor communication between patients and healthcare providers. Those acts, as said earlier, contribute greatly to non-adherence leading to massive drug resistance in future episodes [11, 20]. Malaria often occurs coincidentally with other diseases which often leads patients to take multiple drugs. Pill burden has been reported as a hindrance to adherence [36] because many of these drugs have different schedules, and side effects, this could influence the adherence of AL treatment [4]. However, in this study taking the 1^st^ dose of AL at dispensary showed no significant association unlike findings from Zambia, Malawi, and Tanzania that reported that it had a significant association [20, 37, 38]. This disparity could be due to that in our study only fifteen percent of patients received the first dose of AL at the dispensary whereas more than half of patients in the study of Zambia, Malawi, and Tanzania did.

## Limitations

Recall bias has been introduced in the present study since the generated data also relied on patients’ opinion and self report [11]; they did not know the appropriate time for taking each dose; however, the study tried to minimize the recall bias using data collected within two days after completing the treatment.

Those who did not show blister packs could have thrown them away because they had finished treatment or could have been reluctant to show us the packs because pills were left over that means patients would be more likely to be unwilling to report missed doses [39]; this may lead to overestimation of adherence to AL. Potential determinants of AL adherence like distance to nearby health facilities and waiting time at health facilities were not included in the present study.

## Strengths

We have used two different methods to measure adherence: the pill count (observation of the blister pack) and interview using a systematic questionnaire.

In previous studies patients who had vomited during the course of treatment were excluded. However, our study used it as one criterion to assess adherence status since the national guideline indicated that dose must be repeated if vomited within one hour of ingestion [4].

Furthermore, most previous studies assessed adherence status of patients before the final dose was supposed to be ingested. However; this study assessed the day after all the doses were supposed to be ingested (on day 4) which could get a more accurate assessment.

## Conclusion

Adherence to Artemether–lumefantrine treatment in the current study was low. Children aged < 5 years, and being treated in health post were determinants of AL adherence whereas illiteracy, didn’t know the consequence of discontinued the drug, and had concomitant treatments were factors that hindered the AL adherence treatment.

Stakeholders should emphasize designing appropriate strategies including educational interventions to avert the AL non-adherence and its consequences on drug resistance.

Specifically, malaria patients should collect additional drugs from nearby health facilities if some doses have been vomited and they should strictly stick to AL dispensing information that is given by healthcare providers. Health facilities workers should provide patient-centered counseling and advice during the process of dispensing of AL. Further research should be conducted to evaluate the drug resistance to AL.

## Data Availability

The data set used and/or analyzed during the current study is available from the corresponding author on reasonable request.

## Abbreviations

ACT: Artemisinin combination therapy
AL: Artemether–lumefantrine
ITNS: Insecticide treated nets
MDGs: Millennium development goals
MoH: Ministry of Health
PF: Plasmodium falciparum
PV: Plasmodium vivax
RDT: Rapid diagnostic test
SP: Sulfadoxine-pyrimethamine
TRHB: Tigray Regional Health Bureau
WHO: World Health Organization
